# Differential Role of gp130-Dependent STAT and Ras Signalling for Haematopoiesis Following Bone-Marrow Transplantation

**DOI:** 10.1371/journal.pone.0039728

**Published:** 2012-06-22

**Authors:** Daniela C. Kroy, Lisa Hebing, Leif E. Sander, Nikolaus Gassler, Stephanie Erschfeld, Sara Sackett, Oliver Galm, Christian Trautwein, Konrad L. Streetz

**Affiliations:** 1 Department of Medicine III, University Hospital Aachen, Aachen, Germany; 2 Institute of Pathology, University Hospital Aachen, Aachen, Germany; 3 Department of Medicine IV, University Hospital Aachen, Aachen, Germany; 4 Department of Infectious Diseases and Pulmonary Medicine, Charité University Hospital Berlin, Berlin, Germany; Instituto Nacional de Câncer, Brazil

## Abstract

**Introduction:**

Bone marrow transplantation (BMT) is a complex process regulated by different cytokines and growth factors. The pleiotropic cytokine IL-6 (Interleukin-6) and related cytokines of the same family acting on the common signal transducer gp130 are known to play a key role in bone marrow (BM) engraftment. In contrast, the exact signalling events that control IL-6/gp130-driven haematopoietic stem cell development during BMT remain unresolved.

**Methods:**

Conditional gp130 knockout and knockin mice were used to delete gp130 expression (gp130^ΔMx^), or to selectively disrupt gp130-dependent *Ras* (gp130^ΔMxRas^) or STAT signalling (gp130^ΔMxSTAT^) in BM cells. BM derived from the respective strains was transplanted into irradiated wildtype hosts and repopulation of various haematopoietic lineages was monitored by flow cytometry.

**Results:**

BM derived from gp130 deficient donor mice (gp130^ΔMx^) displayed a delayed engraftment, as evidenced by reduced total white blood cells (WBC), marked thrombocytopenia and anaemia in the early phase after BMT. Lineage analysis unravelled a restricted development of CD4(+) and CD8(+) T-cells, CD19(+) B-cells and CD11b(+) myeloid cells after transplantation of gp130-deficient BM grafts. To further delineate the two major gp130-induced signalling cascades, *Ras*-MAPK and STAT1/3-signalling respectively, we used gp130^ΔMxRas^ and gp130^ΔMxSTAT^ donor BM. BMT of gp130^ΔMxSTAT^ cells significantly impaired engraftment of CD4(+), CD8(+), CD19(+) and CD11b(+) cells, whereas gp130^ΔMxRas^ BM displayed a selective impairment in early thrombopoiesis. Importantly, gp130-STAT1/3 signalling deficiency in BM grafts severely impaired survival of transplanted mice, thus demonstrating a pivotal role for this pathway in BM graft survival and function.

**Conclusion:**

Our data unravel a vital function of IL-6/gp130-STAT1/3 signals for BM engraftment and haematopoiesis, as well as for host survival after transplantation. STAT1/3 and ras-dependent pathways thereby exert distinct functions on individual bone-marrow-lineages.

## Introduction

IL-6 type cytokines, especially IL-6, IL-11, OSM (oncostatin M) and LIF (leukaemia inhibitory factor) in conjunction with their shared common receptor subunit gp130 (glycoprotein 130) play an important role for the regulation of organism homeostasis [1]. Gp130 thereby acts as the membrane bound part of the receptor complex, that upon homo- or hetero-dimerization induces the phosphorylation of its intracellular tyrosines [2] activating *Ras*- or STAT1/3- dependent signalling cascades. The general importance of gp130 and dependent processes becomes evident already during the phase of embryonic development. Gp130 knockout embryos suffer from a severely restricted haematopoiesis as well as from an impaired hepatic development and subsequently die *in utero* [3]. This functionally distinguishes gp130 from its multiple ligands, which show redundant biological functions. Therefore, the phenotype of individual cytokine-knockout mice is often more subtle and less severe than deletion of their receptors.

Introduction of conditional gp130-knockout mice by Betz *et al* [3] provided initial insights into the regulatory role of gp130 in haematopoiesis. Those mice showed a spontaneous mild thrombocytopenia, while leukocyte numbers were increased. The relative amount of T-cells was also reduced. After the induction of chemically induced BM injury those mice showed a delayed recovery in erythrocytes and platelets together with a 40% reduction of haematopoietic progenitors. Another study demonstrated that gp130 is required to maintain the self-renewal capacity of haematopoietic stem cells [4].

In contrast, mice overexpressing the gp130 ligands IL-6 or LIF displayed a hyperproliferation of haematopoietic cells and developed splenomegaly, plasmocytosis, thrombocytosis and extramedullar haematopoiesis [5]. It was shown that IL-6 and LIF synergize with IL-3 and stem cell factor (SCF), which are important for the integrity of haematopoietic progenitor cells [6].

In an effort to delineate the dichotomy of gp130-dependent intracellular signalling, that leads to the activation of *Ras*- or STAT-pathways respectively, partial gp130- knockin/knockout mice were created [7]. Mice lacking the 4 distal tyrosine residues of the cytoplasmic domain of gp130, which constitute the STAT1/3 binding sites, showed elevated numbers of BM precursors and reduced platelet counts [8]. On the contrary, mice carrying a point mutation in gp130 tyrosine-757 display elevated basal STAT3 activation but are deficient for gp130-*Ras*-signalling [9]. These mice develop a broad spectrum of haematopoietic abnormalities, including splenomegaly, lymphadenopathy, and thrombocytosis [10].

Whereas the role of gp130-signals in steady state haematopoiesis has been well characterized, its functions in BMT remain unclear. There is evidence, that gp130 is important in endothelial cells of BM recipient mice [11], yet the more pressing issue, whether it plays a role within the donor cell compartment is still unresolved. This is especially important since donor cells could be easily analysed and conditioned prior to transplantation. This prompted us to carefully dissect the function of gp130 in BM donor cells during the process of engraftment and proliferation.

We have recently demonstrated the contribution of the different gp130-dependent signalling pathways for the maintenance of hepatocyte integrity during liver regeneration and fibrosis development [12]. Here, we identify gp130-STAT1/3 as a vital pathway in leucopoiesis, whereas gp130-*Ras* controls early thrombopoiesis after BMT.

## Materials and Methods

### Animals

Mice were housed in 12-hour light/dark cycles, with free access to food and water and were treated in accordance with the criteria of the German administrative panel on laboratory animal care and approved by the local Animal Care Committee (Landesamt für Natur, Umwelt und Verbraucherschutz Nordrhein-Westfalen, LANUV, NRW, PF 101052, 45610 Recklinghausen, Germany, AZ: 9.93.2.10.35.07.155).

At least 5 animals were analysed per time point. All experiments were repeated at least three times.

C57/BL6/J mice carrying loxP sites flanking exon 16 coding for the gp130 transmembrane domain were crossed with transgenic (tg) mice expressing Cre-recombinase as described previously [3], [13]. Type-I Interferon (IFN)-sensible Mx1 promotor (MxCre) controlled cre-recombinase expression [3]. This was activated by intraperitoneal injection of 100 μg of poly (I: C) (Sigma-Aldrich) 10 and 5 days before the start of the experiment. IFNγ-induced activation of the Mx1 promoter led to the expression of Cre and subsequent deletion of gp130-exon 16. Animals that were negative for the Cre-allele but carried loxP sites in both gp130 alleles (gp130^loxP/loxP^) served as controls and were treated equally.

BM chimeric mice were generated by transplanting freshly isolated BM from GFP transgenic (β-actin/GFP) donors. Wildtype (gp130^loxP/loxP^) or gp130 knockout (gp130^ΔMx^) animals were both injected with poly (I: C) as described above at day 10 and day 5 before used as donor mice. Recipient mice were also pretreated with poly (I: C) followed by a whole body irradiation with 12 Gy. The transplanted animals received antibiotic (Borgal, Schering-Plough, Muenchen-Neuperlach, Germany) containing drinking water for 14 days.

To trace transplanted BM cells, we generated gp130^ΔMxSTAT^ and gp130^ΔMxRas^ GFP-double transgenic mice. Gp130^ΔMxSTAT^ mice were generated by breeding MxCre gp130^loxP/loxP^ with gp130^ΔSTAT/ΔSTAT^ knockin mice expressing a truncated gp130 knockin allele that lacks the essential region for the activation of STAT1 and-3 signalling [9] [14]–[15]. Gp130^ΔMxRas^ mice were generated by crossing MxCre gp130^loxP/loxP^ with gp130^Y757F/Y757F^ knockin mice, which express a gp130 allele carrying a point mutation at tyrosine Y757 thus being defective in Ras-signalling. The genotypes were analysed by PCR for MxCre, gp130^loxP/loxP^, gp130^Y757F^ and the gp130^ΔSTAT^ allele as described previously [16]–[17]. Resulting donor mice were heterozygous for gp130^loxP^ and gp130^Y757F^ or gp130^ΔSTAT^ in conjunction with β-actin/GFP respectively.

A cartoon of the different signalling pathways is displayed in Figure S1A.

### Isolation of cells and flow cytometry

White blood cells (WBC) were counted automatically using an automated cell counter (Hereus, Karlsruhe Germany). After red blood cell lysis (PharmLyse, BD Biosciences, Heidelberg, Germany) cells were stained for CD45, CD11b, CD19, CD4 and CD8 (all eBiosciences, Frankfurt, Germany) and subjected to flow cytometry using a BD Canto II (BD Biosciences, Heidelberg, Germany). Data were analysed using FlowJo software (TreeStar, Ashland, USA).

An example flow cytometry plot for a GFP negative donor mouse (upper graph) as well as for a GFP positive donor animal (lower graph) is shown in Figure S1B. In order to study the absolute numbers of different cell populations, percentages of GFP+ fractions were multiplied with the total number of leucocytes.

Data of donor mice of all different genotypes at 8 weeks of age as well as pI; pC induced donor mice as comparison to transplanted mice is presented in Figure S2A–G.

### In vitro stimulation of freshly isolated BM cells

Freshly isolated BM cells (5×10^6^) of gp130^loxP^, gp130^ΔMx^, gp130^ΔMxRas^ and gp130^ΔMxSTAT^ mice were stimulated with recombinant IL-6 (100 mg/ml) for 2 and 6 hours. Western Blot analysis was performed for unstimulated (control) and stimulated cells.

### SDS Page and Western Blot

For primary antibody incubation, membranes were probed with anti-phospho-STAT-3 (Tyr705; #9131s; Cell Signalling, Frankfurt, Germany) and anti-GAPDH (4699–9555, Poole, Dorset, UK) antibodies. As a secondary antibody, HRP-linked anti-rabbit immunoglobulin G (#7074; Cell Signaling, Frankfurt, Germany) and HRP-linked anti-mouse immunoglobulin G (sc-2005, Santa Cruz, Heidelberg, Germany) were used. The antigen-antibody complexes were visualised using the ECL Chemiluminescence Kit (GE Healthcare, Buckinghamshire, England).

### Statistics

All numerical results are expressed as mean +/− SE and represent data from at least 5 animals per time point. All significant p-values were measured by student's T-test. A value of p<0.05 was considered significant (*  =  p<0.05, **  =  p<0.01, ***  =  p<0.001). Survival curve statistical analysis was performed by a Mantel-Cox test in GraphPad Prism©).

## Results

### Gp130 in donor cells is required for optimal engraftment after BM transplantation (BMT)

**Figure 1:**
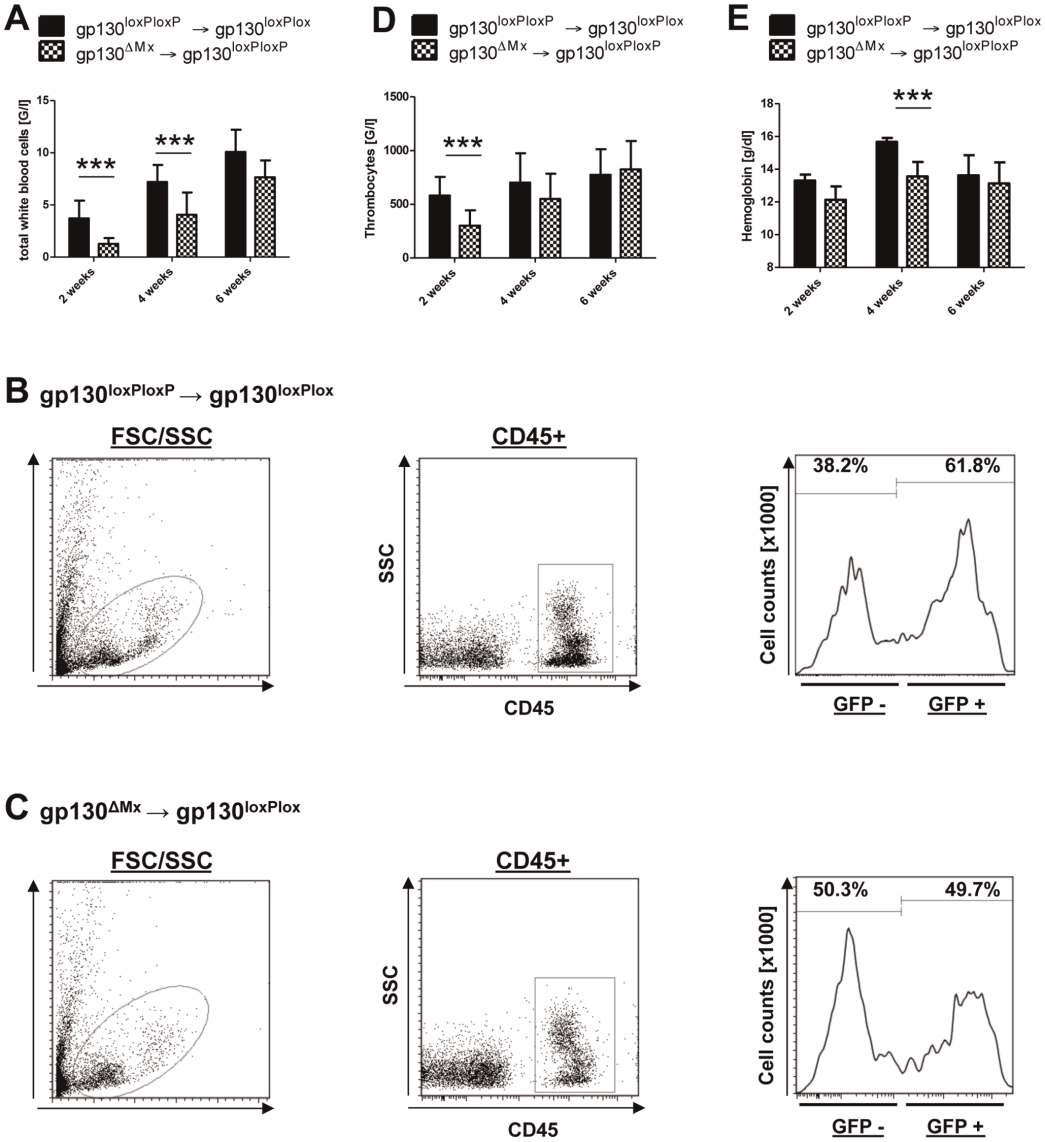
Delayed engraftment of gp130^ΔMx^ donor BM in the early phase after BM transplantation (BMT). Numbers of total white blood cells (WBC) [G/l] after BMT: Displayed are total white blood cell counts after BM transplantation at the indicated time points (2, 4, 6 weeks). Less total white blood cells can be detected in gp130^loxP/loxP^ mice that were transplanted with GFP(+)gp130^ΔMx^ BM compared to GFP(+)gp130^loxP/loxP^ donor mice. **A**) Engraftment of CD45(+)GFP(+) white blood cells (WBC) [G/l] after BMT: Displayed is an example flow cytometry plot 2 weeks after BMT of gp130^loxPloxP^ BM. Gating on all white blood cells in the FSC/SSC is followed by gating on CD45+ cells. The GFP+ (donor) fraction is shown as a histogram. **B**) Delayed engraftment of CD45(+)GFP(+) white blood cells (WBC) [G/l] after BMT of gp130 deficient BM: An example flow cytometry plot 2 weeks after BMT of gp130^ΔMx^ BM is demonstrated. Gating on all white blood cells in the FSC/SSC is followed by gating on CD45+ cells. The GFP+ (donor) fraction is shown as a histogram. Comparison to Fig. 1B shows the decreased GFP+, donor derived, cell fraction. **C**) Gp130 deficiency in donor mice leads to thrombocytopenia: Depicted are the platelet counts 2, 4 and 6 weeks after BMT. Gp130^loxP/loxP^ littermates that received GFP(+)gp130^ΔMx^ BM showed a significant thrombocytopenia 2 weeks after BMT. **D**) Haemoglobin values [g/dl] after BM transplantation: Haemoglobin values 2, 4 and 6 weeks after BM transplantation are depicted with a significant difference 4 weeks after BMT. [***p<0.001].

In order to analyse the functional role of gp130 during the process of BMT, lethally irradiated recipient mice received unfractionated GFP(+)gp130^loxP/loxP^ (gp130 competent – functionally equivalent to wildtype) or GFP(+)gp130^ΔMx^ (gp130-deficient) BM cells. The engraftment of transplanted (donor derived  =  GFP+) cells was analysed by determining the relative number of GFP+ cells in recipient animals. Recipients of gp130^ΔMx^ BM showed significantly lower total white blood cell counts 2 and 4 weeks after BM transplantation compared to recipients that were transplanted with gp130^loxP/loxP^ BM (Figure 1A). Analysis of the CD45(+) cell fraction of GFP(+) cells after transplantation proved the delayed engraftment of gp130^ΔMx^ BM in gp130^loxP/loxP^ recipients (Figure 1B, C and Figure S3A). Interestingly, total white blood cell counts after BM reconstitution was independent of the recipients’ gp130 status, meaning that gp130^ΔMx^ or recipients receiving wildtype (gp130^loxP/loxP^) or gp130-deficient (gp130^ΔMx^) BM had equal WBC numbers after BMT (Figure S4A, B). Therefore, throughout the following experiments we focused on gp130-functions in the haematopoietic donor cells and used only gp130^loxP/loxP^ recipients.

Gp130^loxP/loxP^ mice that were transplanted with gp130^loxP/loxP^ BM showed no change in platelet counts (around 600–800 G/l) as compared to untreated wildtype animals (Figure S2C). In contrast, recipients of gp130^ΔMx^ donor BM had significantly less platelets (about 300 G/l) two weeks after BMT (Figure 1D). Additional analysis of haemoglobin values showed a relative temporary anaemia in recipients of gp130^ΔMx^ BM (Figure 1E).

### Lymphocyte and myeloid cell development after BMT requires intact gp130 signalling in donor cells

**Figure 2:**
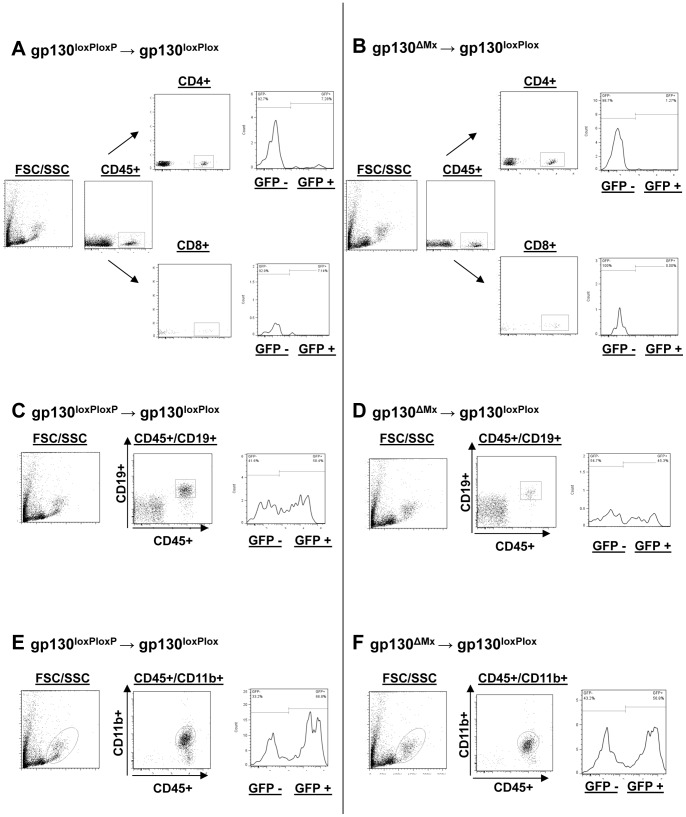
Subgroup analysis of different T-cell subsets after BMT. **A)/B**) Engraftment of CD4(+)/GFP(+) and CD8(+)/GFP(+) T-cells [G/l]: CD4(+)/GFP(+) (upper plots) and CD8(+)/GFP(+) T-cells (lower plots) were analysed by flow cytometry analysis 2, 4 and 6 weeks after BM transplantation. A lower percentage of CD4(+)/GFP(+) and CD8(+)/GFP(+) T-cells could be detected in gp130^loxPloxP^ recipients that were transplanted with GFP(+)gp130^ΔMx^ donor BM. Displayed are example flow cytometry plots for a recipient mouse of wildtype donor BM (A) as well as for a recipient animal of gp130 deficient BM (B) 2 weeks after BMT. **C)/D**) Engraftment of CD19(+)/GFP(+) B-cells [G/l]: CD19(+)/GFP(+) B-cells derived from GFP(+)gp130^ΔMx^ donor BM (Fig. 2D) engrafted decelerated compared to GFP(+)gp130^loxPloxP^ donor BM (Fig. 2C) 2 and 4 weeks after BM transplantation. Shown are example flow cytometry plots 2 weeks after BMT. **E)/F**) Engraftment of CD11b(+)/GFP(+) cells [G/l]: 2 weeks after BM transplantation the percentage of CD11b(+)/GFP(+) cells was lower in gp130^loxP/loxP^ recipients transplanted with GFP(+)gp130^ΔMx^ donor BM compared to controls. Displayed are example flow cytometry plots for 2 weeks transplanted mice having received wildtype (E) or gp130 deficient (F) BM respectively.

Next, we delineated the effects of gp130-signals on immune cell development and differentiation. First, we investigated the development of mature T-cells. Interestingly, we found significantly reduced numbers of CD4(+)/GFP(+) and CD8(+)/GFP(+) T-cells two and four weeks after transplantation of gp130^ΔMx^ BM, compared to transplantation of gp130^loxP/loxP^ cells (Figure 2A, B and Figure S3B, C), demonstrating a severely impaired T-cell development in the absence of functional gp130 in haematopoietic progenitors.

To investigate the engraftment of B-cells we analysed CD19(+)/GFP(+) cell frequencies and numbers. Recipients that were transplanted with gp130^loxP/loxP^ BM had robust B-cell numbers (2–2.5 G/l), which is comparable to untreated animals (Figure S2F). In contrast, mice receiving gp130^ΔMx^ BM showed significantly reduced CD19(+)/GFP(+) B-cells for up to week 4 after transplantation (Figure 2C, D and Figure S3D).

As a marker of myeloid cell development we measured CD11b(+)GFP(+) cells numbers. Interestingly, 2 weeks after BMT we could detect significantly lower CD11b(+) numbers in mice that received gp130^ΔMx^ BM compared to gp130^loxP/loxP^ donors (Figure 2E, F and Figure S3E), suggesting that gp130-signalling is also involved in the engraftment and proliferation of this lineage.

Overall, this lineage specific analysis revealed a pan-leukocyte development and/or engraftment blockade of gp130-deficient BM grafts.

### Differential role of gp130-STAT1/3 and gp130-*Ras* signals during BM engraftment

Intracellular signalling induced by IL-6/gp130 leads to the activation of the JAK/STAT and Ras-pathway. To further evaluate the exact contribution of either pathway in the process of BM transplantation, mice with deficient *Ras* (gp130^ΔMxRas^) or STAT (gp130^ΔMxSTAT^) signalling were used as BM donors. Recipient gp130^loxP/loxP^ mice were analysed 2, 4 and 6 weeks after BM transplantation according to the previous experiments.

**Figure 3:**
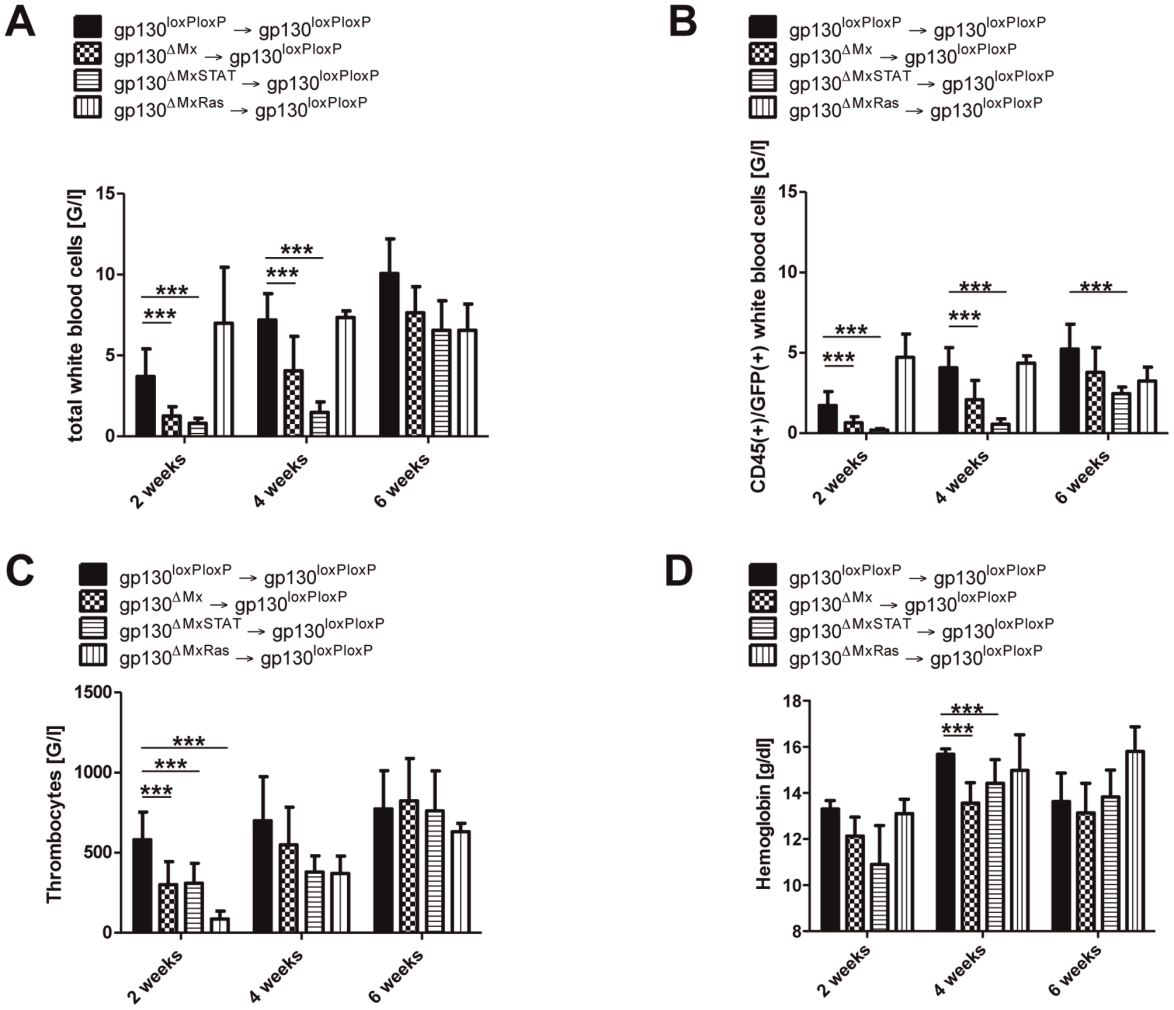
Dissection of intracellular gp130 signalling pathways. **A**) Number of total white blood cells (WBC) [G/l] after BMT: Displayed are the total white blood cell counts after BM transplantation at the indicated time points (2, 4 and 6 weeks). Less total white blood cells could be detected in gp130^loxP/loxP^ mice that were transplanted with GFP(+)gp130^ΔMxSTAT^ BM. Transplantation of GFP(+)gp130^ΔMxRas^ donor BM did not result in any significant difference concerning the total WBC count. **B**) Delayed engraftment of CD45(+)/GFP(+) white blood cells (WBC) [G/l] after BMT is STAT-dependent: Displayed are the CD45(+)/GFP(+) (donor derived) cells after BM transplantation at the indicated time points (2, 4 and 6 weeks). Whereas transplantation of GFP(+)gp130^ΔMxRas^ donor BM into gp130^loxP/loxP^ animals did not lead to a delayed engraftment of CD45(+)/GFP(+) cells, transplantation of gp130^ΔMxSTAT^ into gp130^loxP/loxP^ resulted in a significant decrease of CD45(+)/GFP(+) cells. **C**) Defective Ras and STAT signalling in donor mice leads to thrombocytopenia: Depicted are the platelet counts 2, 4 and 6 weeks after BMT. Transplantation of GFP(+)gp130^ΔMxRas^ as well as GFP(+)gp130^ΔMxSTAT^ donor BM led to a significant thrombocytopenia 2 weeks after BM transplantation. **D**) STAT-deficiency in donor mice leads to anaemia after BM transplantation: Depicted are the haemoglobin values 2, 4 and 6 weeks after BM transplantation with a significant anaemia in gp130^loxP/loxP^ recipients transplanted with GFP(+)gp130^ΔMxSTAT^ donor BM 4 weeks after BMT. [**p<0.01, ***p<0.001].

First, total leukocyte cell counts were determined at different time points after BMT. Interestingly, two and four weeks after BM transplantation mice transplanted with gp130^ΔMxSTAT^ BM displayed very low levels of total white blood cells whereas animals transplanted with gp130^ΔMxRas^ BM showed comparable cell numbers as recipients of gp130^loxP/loxP^ BM (Figure 3A). If anything, gp130^ΔMxRas^ BM reconstituted mice showed enhanced WBC recovery. This observation is in line with previously reported over-activation of STAT3 in gp130^ΔMxRas^ cells [9], [10] (Figure S5).

The delayed engraftment of gp130^ΔMxSTAT^ BM could be corroborated by analysing CD45(+)GFP(+) cells. Mice transplanted with gp130^ΔMxSTAT^ BM displayed less CD45(+)GFP(+) cells than recipients of gp130^ΔMxRas^ or gp130^ΔMx^ BM (Figure 3B).

In contrast to WBC, whose development strongly depended on gp130-STAT1/3 signalling, we found significantly lower platelet counts in animals that were transplanted with either gp130^ΔMxRas^ or GFP(+)gp130^ΔMxSTAT^ BM after 2 weeks. Recipients of gp130^ΔMxRas^ cells displayed a temporarily aggravated thrombocytopenia (Figure 3C). Thus, in contrast to leucopoiesis, which requires gp130-STAT1/3 activation, early thrombopoiesis is mediated by gp130-*Ras* signals.

To complete our blood count analysis, we analysed hemoglobin values after BMT and observed a significant, albeit mild anemia in gp130^ΔMx^ and gp130^ΔMxSTAT^ BM transplanted mice compared to recipients of wildtype cells 4 weeks after BMT (Figure 3D).

### Gp130/STAT-signalling controls development of CD4(+), CD8(+) and CD19(+) cells

**Figure 4:**
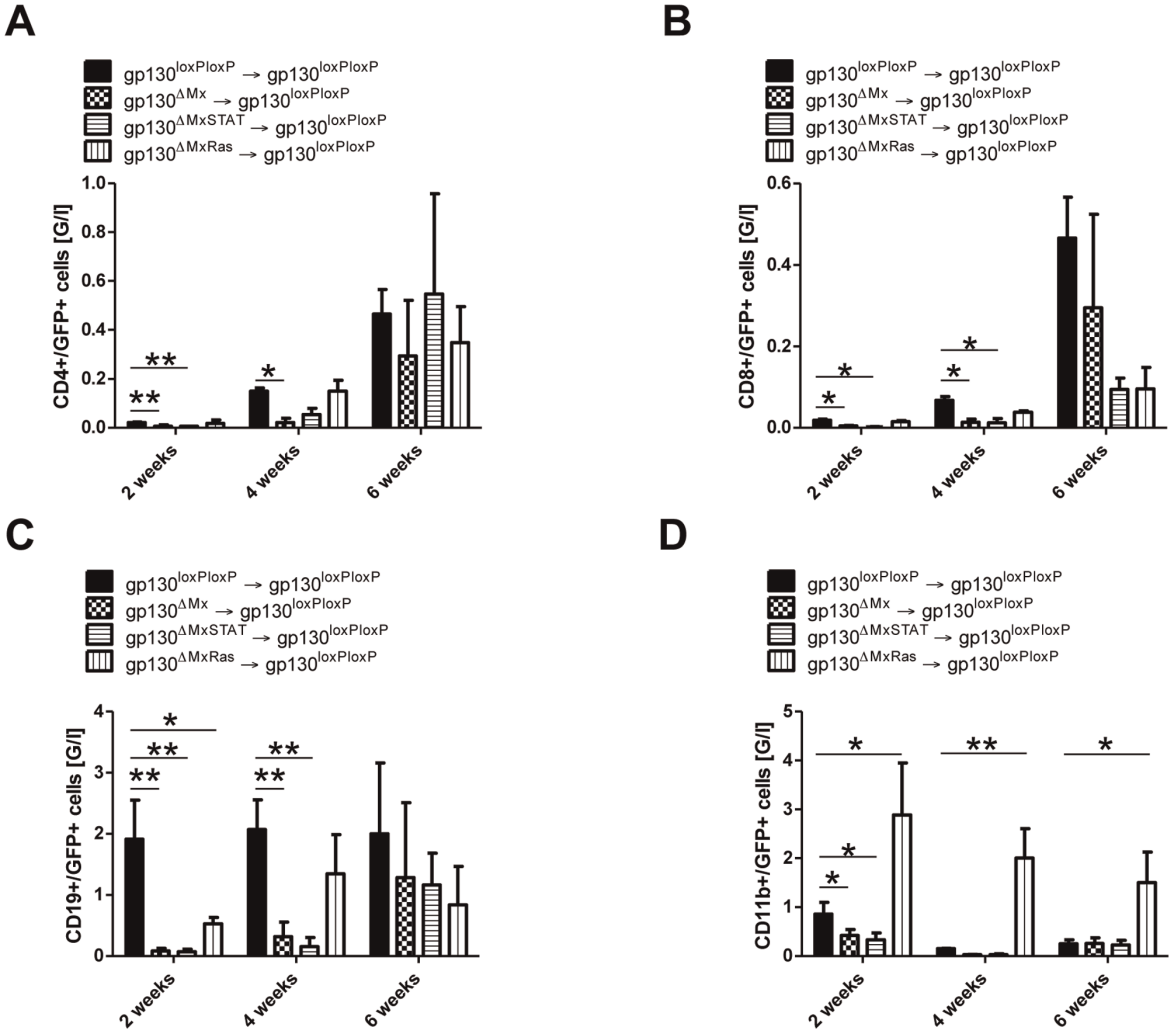
Subgroup analysis of different T-cell subsets after BMT. **A**) Engraftment of CD4(+)/GFP(+) T-cells [G/l]: CD4(+)/GFP(+) T-cells were analysed by flow cytometry analysis 2, 4 and 6 weeks after BM transplantation. A significant lower number of CD4(+)/GFP(+) T-cells could be detected in gp130^loxPloxP^ recipients that were transplanted with GFP(+)gp130^ΔMxSTAT^ donor BM. Transplantation of GFP(+)gp130^ΔMxRas^ donor BM resulted in the same number of CD4(+)/GFP(+) T-cells as transplantation of gp130^loxP/loxP^ BM into gp130^loxP/loxP^ littermates. **B**) Engraftment of CD8(+)/GFP(+) T-cells [G/l]: The absolute number of CD8(+)/GFP(+) T-cells was determined by flow cytometry analysis 2, 4 and 6 weeks after BM transplantation. The number of CD8(+)/GFP(+) T-cells was significantly lower in wildtype mice that were transplanted with GFP(+)gp130^ΔMxSTAT^ 2 and 4 weeks after BMT. **C**) Engraftment of CD19(+)/GFP(+) B-cells [G/l]: CD19(+)/GFP(+) B-cells derived from GFP(+)gp130^ΔMxSTAT^ donor BM engrafted into recipient mice with significant differences at the 2 and 4 week time point. Gp130^loxP/loxP^ recipients of GFP(+)gp130^ΔMxRas^ donor BM also displayed a somewhat delayed engraftment 2 weeks after BMT but recovered faster as shown 4 and 6 weeks after BMT. **D**) Engraftment of CD11b(+)/GFP(+) cells [G/l]: Transplantation of GFP(+)gp130^ΔMxRas^ donor BM into gp130^loxP/loxP^ resulted in a significant increase of CD11b(+)/GFP(+) cells at all indicated time points (2, 4, 6 weeks) after BMT. GFP(+)gp130^ΔMxSTAT^ donor BM led to the same number of CD11b(+)/GFP(+) cells as the transplantation of GFP(+)gp130^ΔMx^ donor BM. [*p<0.05, **p<0.01].

Given the central role for gp130 for CD4(+) and CD8(+), T cell, CD19(+) B cell and CD11b(+) myeloid cell development after BMT (Figure 2, Figure S3), we next aimed to delineate the responsible gp130-dependent intracellular signalling pathways in donor cells. In line with the observed gp130-STAT1/3-dependency of WBC recovery after BMT, we found that mice transplanted with gp130^ΔMxSTAT^ BM showed the similarly reduced numbers of CD4(+)/GFP(+), CD8(+)/GFP(+), CD11b(+)/GFP(+) and CD19(+)GFP(+) cells as mice transplanted with complete gp130 deficient (gp130^ΔMx^) BM (Figure 4A-D). In contrast, animals receiving gp130^ΔMxRas^ BM showed CD4(+)/GFP(+) and CD(8+)/GFP(+) cell numbers comparable to those of recipients of gp130^loxP/loxP^ BM (Figure 4A, B). Of note, two weeks after BMT B-cell engraftment and proliferation as determined by CD19(+)/GFP(+)cells was significantly delayed in both gp130^ΔMxSTAT^ and gp130^ΔMxRas^ donor groups (Figure 4C). Recovery however was significantly faster in mice receiving gp130^ΔMxRas^ BM. The most striking phenotype was observed for CD11b(+) cell recovery. Here, gp130- and gp130-STAT1/3 deficiency delayed engraftment, whereas gp130^ΔMxRas^ donor cells displayed a strikingly overshooting response with up to five-fold increase in circulating CD11b(+)GFP+(+) numbers compared to all other groups (Figure 4D). This result indicates, that while gp130-STAT1/3 signalling is not absolutely required for engraftment of CD11b(+) cells, its overactivation (as observed in gp130^ΔMxRas^ cells, Figure S2) represents a very potent stimulus for myelopoiesis after BMT.

### Gp130 dependent STAT-signalling in transplanted cells is essential for survival after BMT

**Figure 5:**
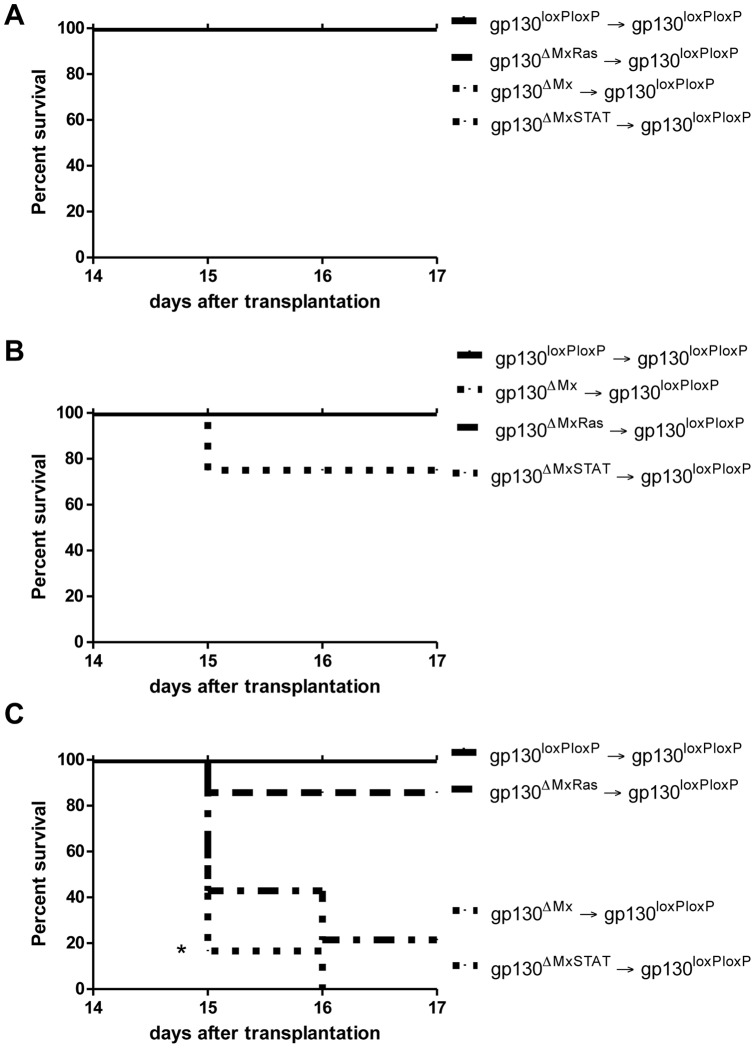
Survival analysis after BM transplantation. A) Survival curve after BMT of 1×10^6^ donor cells (6 mice per group): Recipient mice of all different donor genotypes survived BMT of 1×10^6^ donor cells. B) Survival curve after BMT of 2×10^5^ donor cells (8 mice per group): gp130^ΔMxSTAT^ transplanted mice showed a 75% survival after BMT with 2×10^5^ donor cells. All the other animals survived BMT to 100%. C) Survival curve after BM transplantation using the amount of 5×10^4^ donor cells (6 mice per group): Transplantation of gp130^loxP/loxP^ donor BM into gp130^loxP/loxP^ recipient mice led to a survival rate of 100%. 75% of gp130^loxP/loxP^ recipients of gp130^ΔMxRas^ donor BM survived the experiment. However, if gp130^loxP/loxP^ recipients were transplanted with gp130^ΔMx^ BM, they survived in 33%. Finally, transplantation of gp130^ΔMxSTAT^ donor BM led to 100% mortality with no (0%) surviving recipient mice. [*p<0.05].

To unravel whether the differences in gp130 dependent intracellular signalling during cellular engraftment represent a limiting step for BMT we performed a survival analysis. To this end we determined the critical threshold for host survival by transplanting decreasing numbers of BM cells into lethally irradiated animals (Fig. 5). Whereas transplantation of 10^6^ unfractionated donor cells resulted in 100% survival – regardless of donor BM genotype (Figure 5A), 2×10^5^ donor cells induced 25% mortality solely in the group of gp130^ΔMxSTAT^ donors (Figure 5B). Finally, we further reduced the number of transplanted donor cells to 5×10^4^ cells. Under these conditions, none of the mice that received gp130^ΔMxSTAT^ cells survived BMT, while 33% of animals receiving gp130^ΔMx^ and 75% of mice transplanted with gp130^ΔMxRas^ survived (Figure 5C). In contrast, survival was 100% in mice receiving gp130^loxP/loxP^ BM grafts (Figure 5C).

Collectively, these data clearly indicate that gp130-STAT1/3 signals play a predominant role in donor cells after BMT, regulating BM engraftment and importantly, controlling the outcome of BMT.

## Discussion

BMT is a clinically well-established procedure for decades. However, a detailed understanding of underlying molecular processes involved in stem cell engraftment and proliferation is still incomplete. IL-6 is an important cytokine that not only regulates the homeostasis of haematopoietic stem cells but that is prominently involved in the control of various inflammatory processes [18]. The latter is important especially for BMT, which naturally comprises the need for immunosuppression, thereby provoking many clinically relevant infectious and inflammatory conditions. Moreover, patients who finally undergo BMT have usually been treated before with several bone marrow injuring chemotherapeutic drugs. Due to the toxic nature of most used therapeutics (incl. irradiation) not only the stem cell pool, but also the microenvironment including endothelial and supporting stromal cells in recipient’s BM is affected. Thus, subsequently infused donor cells do not only have to combat with immunological challenges but also with the situation of an inflamed and structurally disintegrated stem cells niche. Interestingly, the regulatory interleukin-6 (IL-6) pathway also seems to play an important role during those pre-conditioning processes [19] and is therefore of particular relevance.

Pathway-specific biological effects for gp130-dependent *Ras*- and STAT- signalling have been demonstrated in a number of tissues including the liver, which as the major regulator of the acute-phase response plays a central role in the control of innate immune functions [20], [21]. In contrast, until now, little was known about contribution of either intracellular signalling pathway following BMT. The aim of the current study was therefore now to delineate gp130-*Ras*- and gp130-STAT-dependent pathways in a clinically relevant *in vivo* mouse model of BMT.

An earlier study using mice with an endothelial cell specific gp130-deletion using a Tie2-Cre construct, provided evidence for a spontaneous BM dysfunction [11]. The BM of those mice was hypocellular, while haematopoietic stem cells seemed unaffected. As a consequence, extramedullary haematopoiesis was enhanced under this condition. Further analysis then revealed a functional defect in endothelial stromal cells that led to a dysfunctional microenvironment in Tie2-Cre/gp130 mice, a phenotype that could not be rescued by BMT. In our study we failed to detect a delayed BM engraftment or a disturbed proliferation of any haematopoietic cell population, if gp130^ΔMx^ mice (lacking gp130 in all cells, including endothelial cells) were used as recipients of wildtype BM (Figure S4A, B). However, we would like to point out that the induced gp130-knockout in BM stromal cells and endothelial cells may have been incomplete.

In contrast, we observed a strong dependency on gp130-signals within the transplanted BM cells. Mice receiving gp130-depleted BM showed transient leukopenia, anaemia and thrombocytopenia and a severely compromised survival if low cell numbers were transferred (Figure 1, Figure 5 and Figure S3). Thus, gp130-dependent signalling in donor cells seems to affect all BM lineages (Figure 1, Figure 3). This is an important and novel observation because it demonstrates that gp130-dependent gene activation is required for proper expansion of blood progenitor cell lineage. Notably, 6 weeks after irradiation and transplantation most lineages were able to recover, which may be best explained by compensatory mechanisms such as extramedullary haematopoiesis. Importantly, we did not observe a preferential selection of potentially remaining non-gp130-deleted cells in the BM of recipient mice. This could reflect that gp130 is most important for proliferation of the rather immature BM progenitors. Moreover, very likely there is a threshold effect of gp130, because otherwise the transplantation of gp130-deficient BM would have even more severe effects on survival. The second indication for dose-dependency stems from our experiment using limited amounts of donor cells (Figure 5). Here, recipient mice ultimately died only if the number of transplanted cells was reduced below a certain threshold of approximately 1×10^5^ cells (Figure 5B, C).

Under real life clinical conditions treatment of neoplasms – not only hematopoietic neoplasms – and BM-failure comprises the homeostasis of the whole organism. Both, the disease itself and the used treatments provoke a) a local (in the BM itself) and b) an often even systemic inflammatory environment within the affected patient that is mediated by cytokines or growth factors. Here, IL-6 is relevant as the messenger who controls the hepatic acute phase reaction [20]. Humoral factors secreted from bone-marrow stromal cells as a response to chemotherapeutic drugs or biological treatments also act paracrine on hematopoietic stem cells itself, thereby sometimes sustaining and perpetuating the underlying inflammatory or malignant condition [22]. Those prerequisites make it even more important to understand the biology of bone-marrow regeneration and involved regulating factors.

Here, we now demonstrate that cells with a deficient gp130-STAT-signalling pathway (gp130^ΔMxSTAT^) were acutely compromised in their ability to generate effective numbers of WBCs or CD45(+) cells, respectively (Figure 3A, B). This matches recent studies reporting a STAT3-dependence of T-cell development in patients with autosomal-dominant hyper-IgE syndrome (AD-HIES) [23]. Interestingly, early thrombopoiesis seemed to require gp130-Ras*-*signalling, since recipients of gp130^ΔMxRas^ BM displayed severe thrombocytopenia 2 weeks post BMT. Even more striking was the observed overshooting expansion of CD11b(+) cells after gp130^ΔMxRas^ BMT. As mentioned earlier, cells bearing the gp130-757Y mutation (gp130^ΔMxRas^) are known to spontaneously over activate the gp130-STAT3 pathway (Figure S5). Thus, the increased amount of CD11b(+) could indicate that permanent gp130-STAT3-activation is a potent driver of strong myelopoiesis which has not been previously reported [9]. Therefore, gp130-STAT3 could represent a valuable therapeutic target to treat neutropenic conditions after BMT, although limitations exist as discussed below.

Taken together our data now unravel differential effects of either STAT- or Ras-dependent signalling in BM cells after transplantation. Proper function of the STAT-signalling pathway is most important for graft survival and its disruption can cause severe graft failure. Data about the functionality of this pathway under the condition of BMT in patients are very limited. It would be of interest to determine whether patients with graft failure or under chemotherapy display altered gp130-STAT responses that might contribute to BM dysfunction.

Yet, there is evidence for a general importance of this signalling cascade in the development of hematopoietic malignancies [24]–[25]. Here mutations in the gp130-bound and activated JAK2-gene – which lead to a permanent activation of the STAT pathway and carry the potential of a subsequent malignant cell transformation – are causally related to myeloproliferative neoplasms. This hampers strategies intending to over stimulate the gp130 pathway with activating therapeutics [26]. Therefore further research is necessary to clarify, how eventually a timely limited and only temporary interference with the gp130-STAT pathway might be of benefit for BM regeneration after transplantation or chemotherapy as well as in BM failure syndromes. Moreover, we need to get a deeper general knowledge about the interplay of cytokines and messengers controlling regeneration and engraftment of bone marrow at the stromal/stem cell interface to improve therapeutic alternatives.

## Supporting Information

Figure S1 **A) Cartoon illustrating the used different genotypes: In a wildtype condition, IL-6 binds to its receptor gp80 and forms a complex with gp130 receptor molecules.** This leads to the dimerization of gp130 with its subsequent intracellular phosphorylation. Depending on the phosphorylated tyrosine-residue the downstream signal activates either the STAT or Ras pathway (gp130^loxP/loxP^). Gp130^ΔMx^ mice carry a conditional gp130 knockout, with neither STAT nor Ras signalling cascade activated. Lack of the four distal tyrosines, the essential region for the activation of STAT1/3 signalling is the characteristic of gp130^ΔMxSTAT^ animals. Gp130^ΔMxRas^ mice were generated by crossing MxCre gp130^loxP/loxP^ with gp130^Y757F/Y757F^ knockin mice, which express a gp130 allele carrying a point mutation at tyrosine Y757 thus being defective in Ras-signalling. **B**) Flow cytometry analysis of peripheral blood: Displayed is the flow cytometry analysis of a GFP negative (upper histogram) and a GFP positive (lower histogram) donor mouse. Pre-transplant flow cytometry conditions also served as controls to determine the threshold for GFP positivity after BMT.
(TIFF)

Figure S2 **WBC, haemoglobin, thrombocyte count and subgroup analyses for untransplanted/untreated as well as untransplanted/pI: pC treated 8 week old mice (n = 5 per group).** **A**) WBC counts for all genotypes do not show significant differences pre-transplant. **B**) Haemoglobin values do not differ significantly between different genotypes pre transplant. **C**) No significant differences were detected in thrombocyte levels pre transplant. **D**) CD4 T cell counts did not show significant differences although there was a trend towards more CD4 T cells in untreated gp130^ΔMxSTAT^ animals. **E**) CD8 T cells also tended to be higher in untreated gp130^ΔMxSTAT^ mice although no significant differences were detected. **F**) CD19+ B cells were lower but not significantly decreased in all three genotype groups compared to untreated wildtype littermates. **G**) CD11b+ cells were increased without significance in untreated as well as pI: pC treted wildtype mice compared to the knockin/knockout genotypes.
(TIFF)

Figure S3 **Delayed engraftment of gp130^ΔMx^ donor BM in the early phase after BM transplantation. A**) Delayed engraftment of CD45(+)GFP(+) white blood cells (WBC) [G/l] after BMT: Displayed are CD45(+)GFP(+) (donor derived) cells after BM transplantation at the indicated time points (2, 4, 6 weeks). A delay in the engraftment of GFP(+)gp130^ΔMx^ BM transplanted into gp130^loxP/loxP^ can be demonstrated. **B**) Engraftment of CD4(+)/GFP(+) T-cells [G/l]: CD4(+)/GFP(+) T-cells were analyzed by flow cytometry analysis 2, 4 and 6 weeks after BM transplantation. A significant lower number of CD4(+)/GFP(+) T-cells could be detected in gp130^loxPloxP^ recipients that were transplanted with GFP(+)gp130^ΔMx^ donor BM. **C**) Engraftment of CD8(+)/GFP(+) T-cells [G/l]: The absolute number of CD8(+)/GFP(+) T-cells was determined by flow cytometry analysis 2, 4 and 6 weeks after BM transplantation. Gp130^loxPloxP^ animals that were transplanted with GFP(+)gp130^ΔMx^ donor BM displayed significantly less CD8(+)/GFP(+) T-cells 2 and 4 weeks after BMT compared to GFP(+)gp130^loxP/loxP^ donor mice. **D**) Engraftment of CD19(+)/GFP(+) B-cells [G/l]: CD19(+)/GFP(+) B-cells derived from GFP(+)gp130^ΔMx^ donor BM engrafted decelerated compared to GFP(+)gp130^loxPloxP^ donor BM 2 and 4 weeks after BM transplantation. **E**) Engraftment of CD11b(+)/GFP(+) cells [G/l]: 2 weeks after BM transplantation the number of CD11b(+)/GFP(+) cells was significantly lower in gp130^loxP/loxP^ recipients transplanted with GFP(+)gp130^ΔMx^ donor BM compared to controls. 4 and 6 weeks after BM transplantation both groups show a decreasing number of CD11b(+)/GFP(+) compared to the 2 week time point. [*p<0,05, **p<0,01, ***p<0,001]
(TIFF)

Figure S4 **gp130 status of recipient mice does not affect WBC engraftment after BMT.** **A**) Numbers of total white blood cells (WBC) [G/l] after BMT: Displayed are total white blood cell counts after BM transplantation at the indicated time points (2, 4, 6 weeks). No significant differences could be detected for BMT of gp130^loxP/loxP^ donor BM in wildtype or gp130 deficient recipient mice. **B**) Engraftment of CD45(+)GFP(+) white blood cells (WBC) [G/l] after BMT does not depend on recipient’s gp130 status: Displayed are CD45(+)GFP(+) (donor derived) cells after BM transplantation at the indicated time points (2, 4, 6 weeks). No significant differences could be detected for BMT of gp130^loxP/loxP^ donor BM in wildtype or gp130 deficient recipient mice.
(TIFF)

Figure S5 **Mx-Cre-mediated deletion efficacy of gp130-activation.** In order to demonstrate the Mx-Cre mediated deletion-efficacy in different mouse genotypes, we isolated BM of all used genotypes (gp130^loxPloxP^, gp130^ΔMx^, gp130^ΔMxRas^, gp130^ΔMxSTAT^) after pIpC-injection. 5×10^6^ cells were stimulated with recombinant IL-6 (100 mg/ml), for 2 and 6 hours. Finally, cells were harvested from unstimulated controls as well as stimulated BM cells and analyzed by Western Blot for STAT3-phosphorylation. Gp130loxPloxP mice showed an increased STAT3-phosphorylation after 2 hours, even increasing after 6 hours. In contrast, gp130^ΔMx^ littermates showed much less phosphorylation, reflecting the abolished signalling. Gp130^ΔMxRas^ animals displayed a hyperactivation of STAT3 after IL-6 stimulation and gp130^ΔMxSTAT^ mice also showed a greatly diminished STAT3-phosphorylation.
(TIFF)
