# Supplementary figures and images for: Differential Role of gp130-Dependent STAT and Ras Signalling for Haematopoiesis Following Bone-Marrow Transplantation
Source: PLoS One. 2012 Jun 22;7(6):e39728. doi: 10.1371/journal.pone.0039728 (PMC3382143; doi:10.1371/journal.pone.0039728)

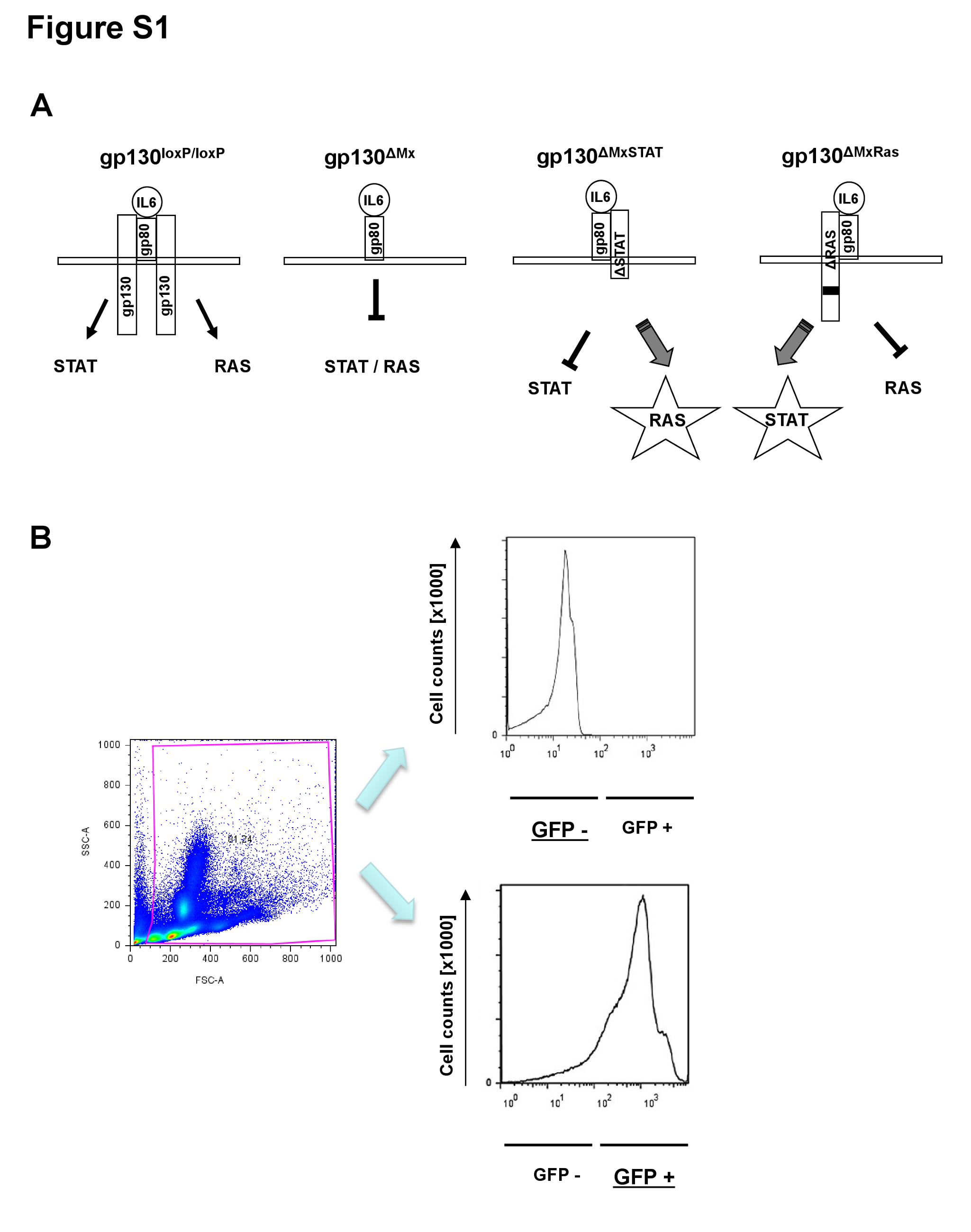

Supplement: Figure S1 — A) Cartoon illustrating the used different genotypes: In a wildtype condition, IL-6 binds to its receptor gp80 and forms a complex with gp130 receptor molecules. This leads to the dimerization of gp130 with its subsequent intracellular phosphorylation. Depending on the phosphorylated tyrosine-residue the downstream signal activates either the STAT or Ras pathway (gp130loxP/loxP). Gp130ΔMx mice carry a conditional gp130 knockout, with neither STAT nor Ras signalling cascade activated. Lack of the four distal tyrosines, the essential region for the activation of STAT1/3 signalling is the characteristic of gp130ΔMxSTAT animals. Gp130ΔMxRas mice were generated by crossing MxCre gp130loxP/loxP with gp130Y757F/Y757F knockin mice, which express a gp130 allele carrying a point mutation at tyrosine Y757 thus being defective in Ras-signalling. B) Flow cytometry analysis of peripheral blood: Displayed is the flow cytometry analysis of a GFP negative (upper histogram) and a GFP positive (lower histogram) donor mouse. Pre-transplant flow cytometry conditions also served as controls to determine the threshold for GFP positivity after BMT. (TIFF) [file pone.0039728.s001.tiff]

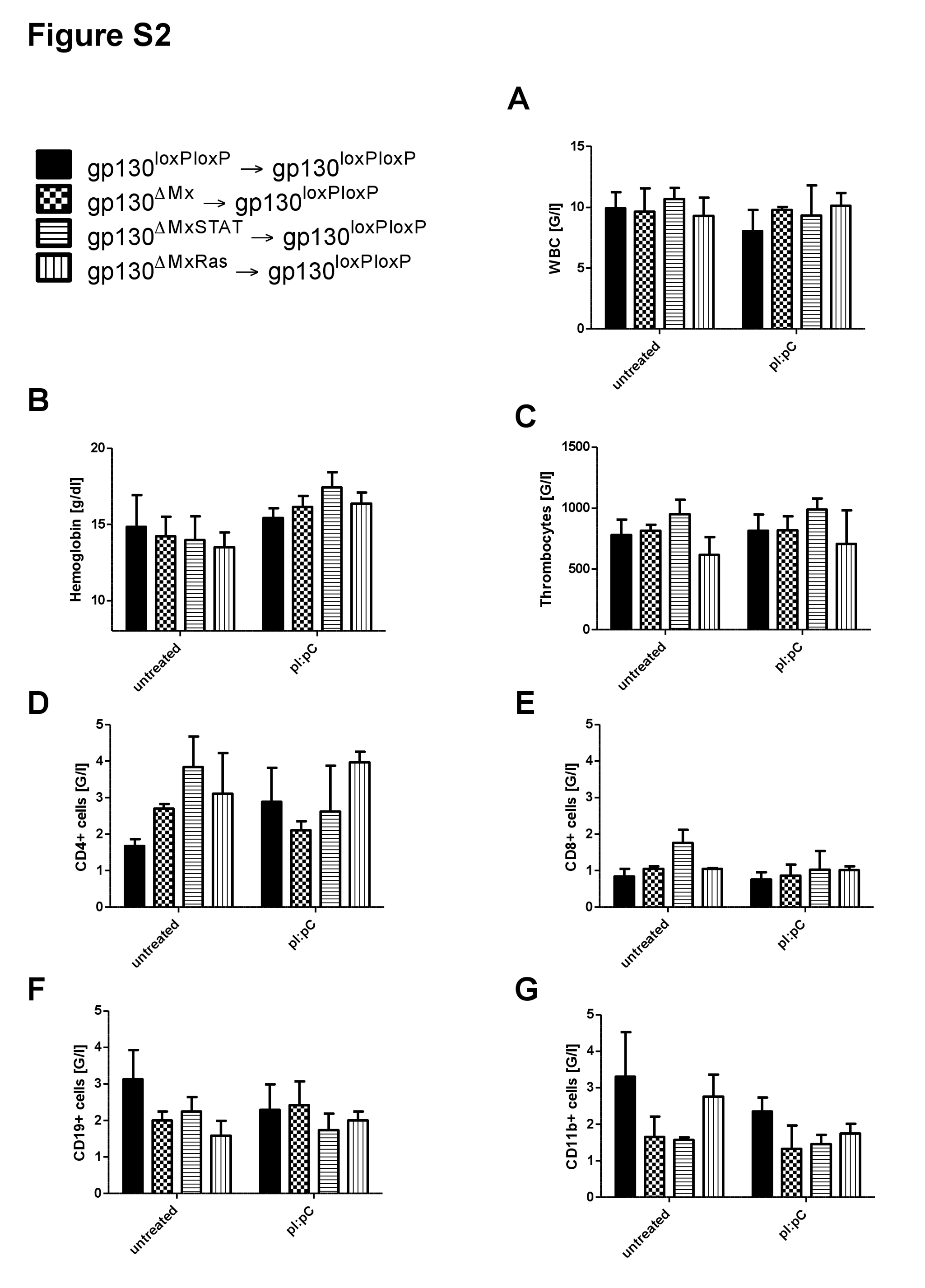

Supplement: Figure S2 — WBC, haemoglobin, thrombocyte count and subgroup analyses for untransplanted/untreated as well as untransplanted/pI: pC treated 8 week old mice (n = 5 per group). A) WBC counts for all genotypes do not show significant differences pre-transplant. B) Haemoglobin values do not differ significantly between different genotypes pre transplant. C) No significant differences were detected in thrombocyte levels pre transplant. D) CD4 T cell counts did not show significant differences although there was a trend towards more CD4 T cells in untreated gp130ΔMxSTAT animals. E) CD8 T cells also tended to be higher in untreated gp130ΔMxSTAT mice although no significant differences were detected. F) CD19+ B cells were lower but not significantly decreased in all three genotype groups compared to untreated wildtype littermates. G) CD11b+ cells were increased without significance in untreated as well as pI: pC treted wildtype mice compared to the knockin/knockout genotypes. (TIFF) [file pone.0039728.s002.tiff]

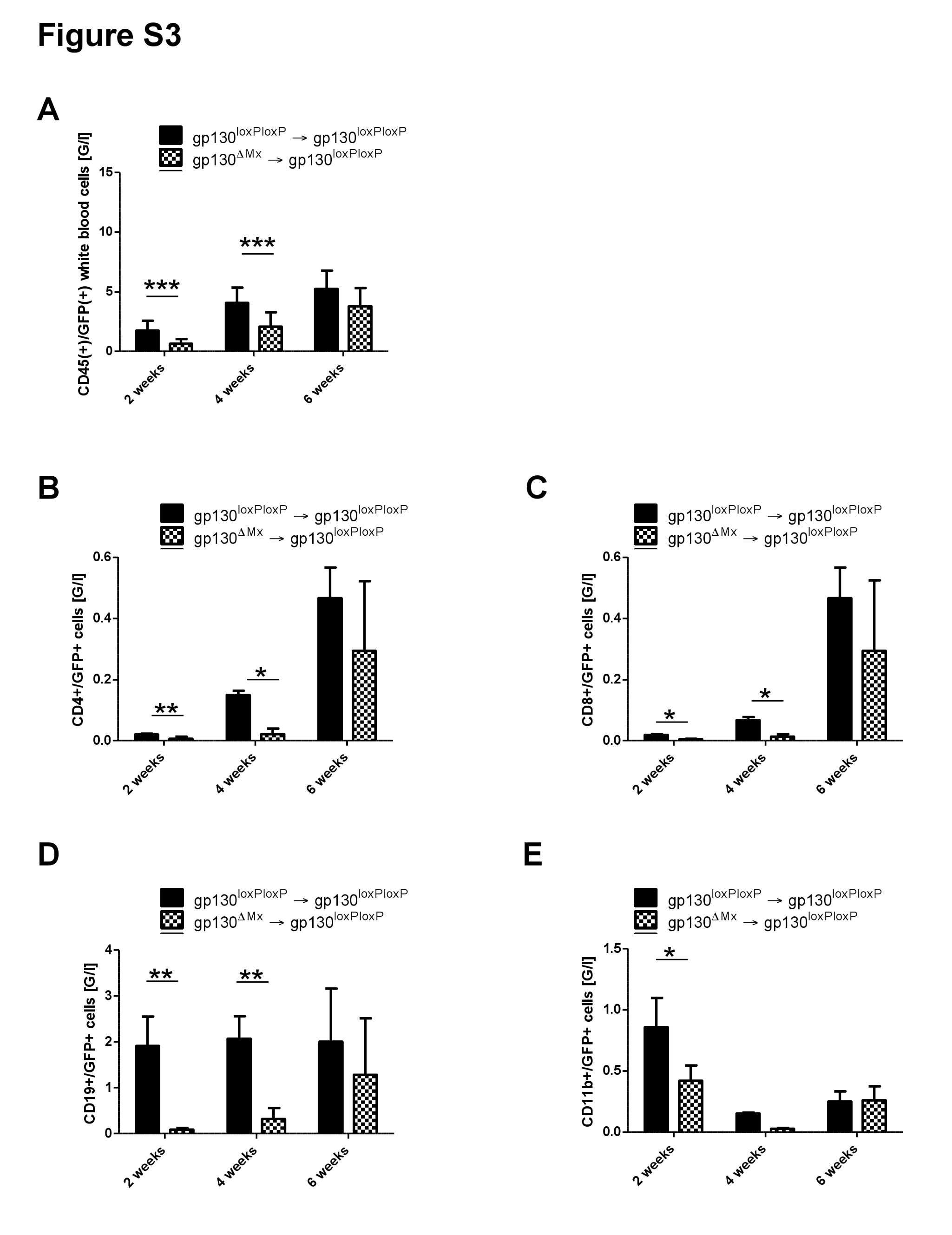

Supplement: Figure S3 — Delayed engraftment of gp130ΔMx donor BM in the early phase after BM transplantation. A) Delayed engraftment of CD45(+)GFP(+) white blood cells (WBC) [G/l] after BMT: Displayed are CD45(+)GFP(+) (donor derived) cells after BM transplantation at the indicated time points (2, 4, 6 weeks). A delay in the engraftment of GFP(+)gp130ΔMx BM transplanted into gp130loxP/loxP can be demonstrated. B) Engraftment of CD4(+)/GFP(+) T-cells [G/l]: CD4(+)/GFP(+) T-cells were analyzed by flow cytometry analysis 2, 4 and 6 weeks after BM transplantation. A significant lower number of CD4(+)/GFP(+) T-cells could be detected in gp130loxPloxP recipients that were transplanted with GFP(+)gp130ΔMx donor BM. C) Engraftment of CD8(+)/GFP(+) T-cells [G/l]: The absolute number of CD8(+)/GFP(+) T-cells was determined by flow cytometry analysis 2, 4 and 6 weeks after BM transplantation. Gp130loxPloxP animals that were transplanted with GFP(+)gp130ΔMx donor BM displayed significantly less CD8(+)/GFP(+) T-cells 2 and 4 weeks after BMT compared to GFP(+)gp130loxP/loxP donor mice. D) Engraftment of CD19(+)/GFP(+) B-cells [G/l]: CD19(+)/GFP(+) B-cells derived from GFP(+)gp130ΔMx donor BM engrafted decelerated compared to GFP(+)gp130loxPloxP donor BM 2 and 4 weeks after BM transplantation. E) Engraftment of CD11b(+)/GFP(+) cells [G/l]: 2 weeks after BM transplantation the number of CD11b(+)/GFP(+) cells was significantly lower in gp130loxP/loxP recipients transplanted with GFP(+)gp130ΔMx donor BM compared to controls. 4 and 6 weeks after BM transplantation both groups show a decreasing number of CD11b(+)/GFP(+) compared to the 2 week time point. [*p<0,05, **p<0,01, ***p<0,001] (TIFF) [file pone.0039728.s003.tiff]

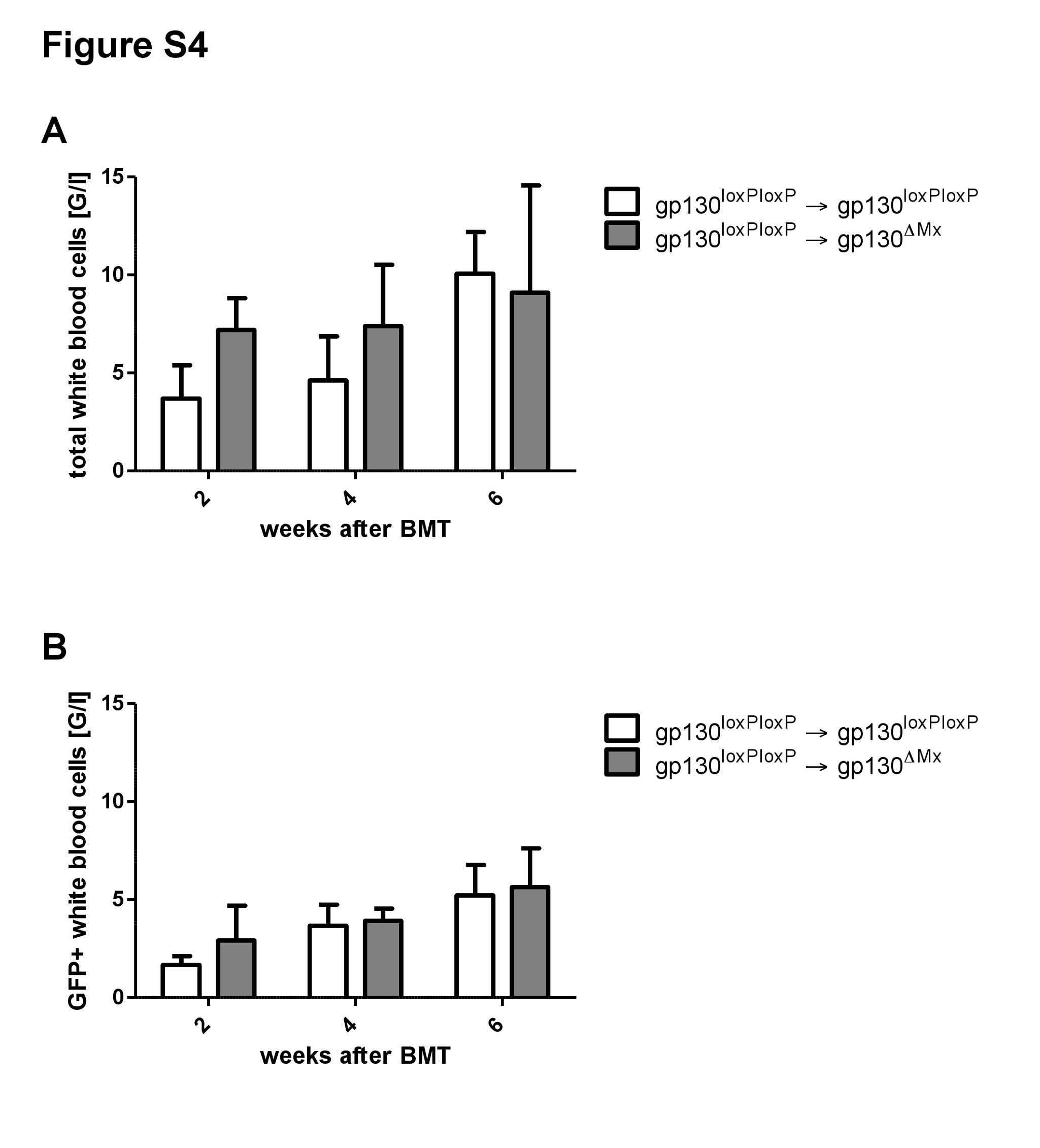

Supplement: Figure S4 — gp130 status of recipient mice does not affect WBC engraftment after BMT. A) Numbers of total white blood cells (WBC) [G/l] after BMT: Displayed are total white blood cell counts after BM transplantation at the indicated time points (2, 4, 6 weeks). No significant differences could be detected for BMT of gp130loxP/loxP donor BM in wildtype or gp130 deficient recipient mice. B) Engraftment of CD45(+)GFP(+) white blood cells (WBC) [G/l] after BMT does not depend on recipient’s gp130 status: Displayed are CD45(+)GFP(+) (donor derived) cells after BM transplantation at the indicated time points (2, 4, 6 weeks). No significant differences could be detected for BMT of gp130loxP/loxP donor BM in wildtype or gp130 deficient recipient mice. (TIFF) [file pone.0039728.s004.tiff]

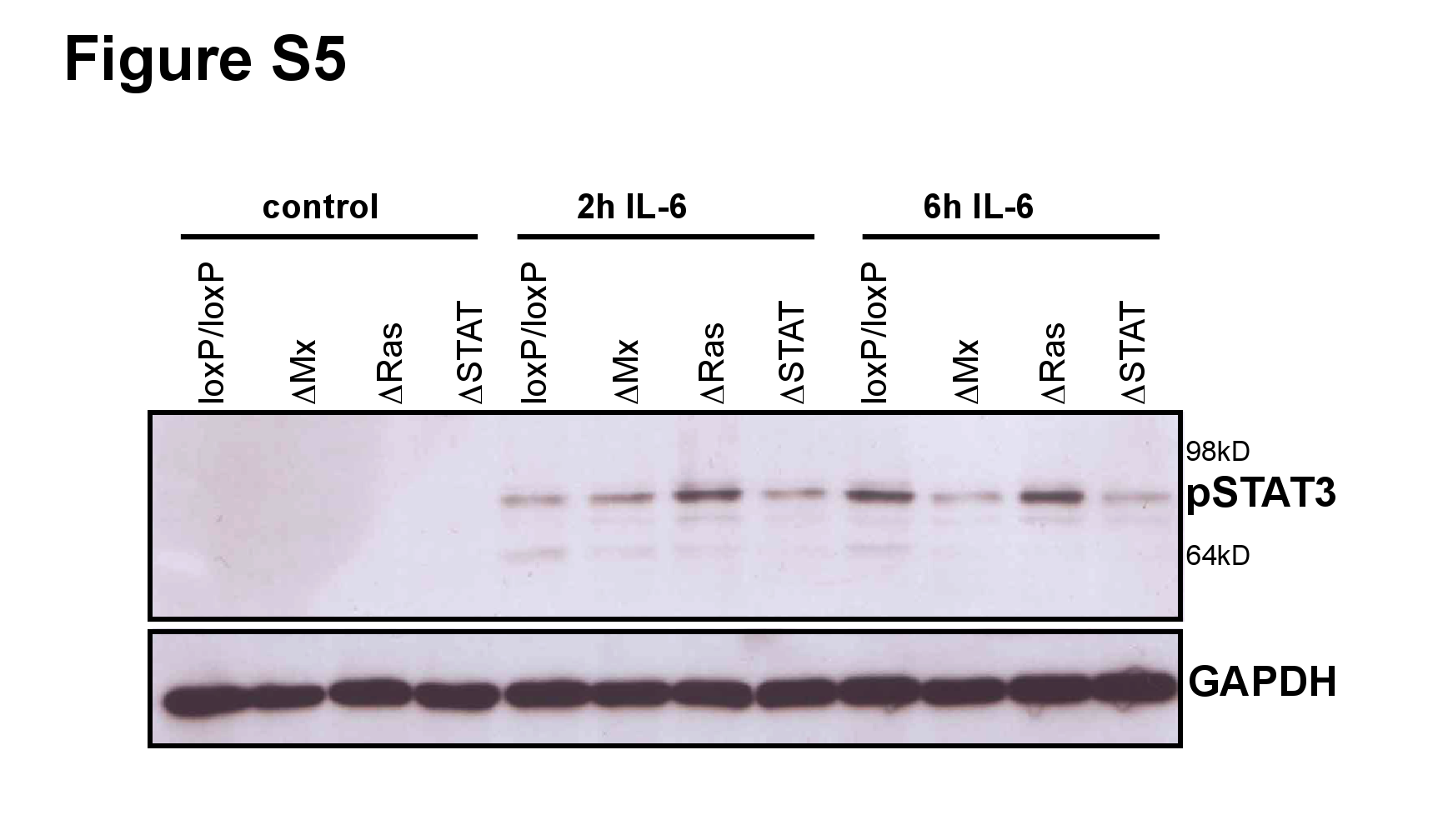

Supplement: Figure S5 — Mx-Cre-mediated deletion efficacy of gp130-activation. In order to demonstrate the Mx-Cre mediated deletion-efficacy in different mouse genotypes, we isolated BM of all used genotypes (gp130loxPloxP, gp130ΔMx, gp130ΔMxRas, gp130ΔMxSTAT) after pIpC-injection. 5×10^6^ cells were stimulated with recombinant IL-6 (100 mg/ml), for 2 and 6 hours. Finally, cells were harvested from unstimulated controls as well as stimulated BM cells and analyzed by Western Blot for STAT3-phosphorylation. Gp130loxPloxP mice showed an increased STAT3-phosphorylation after 2 hours, even increasing after 6 hours. In contrast, gp130ΔMx littermates showed much less phosphorylation, reflecting the abolished signalling. Gp130ΔMxRas animals displayed a hyperactivation of STAT3 after IL-6 stimulation and gp130ΔMxSTAT mice also showed a greatly diminished STAT3-phosphorylation. (TIFF) [file pone.0039728.s005.tiff]
